# Abnormal connectivity in the sensorimotor network predicts attention deficits in traumatic brain injury

**DOI:** 10.1007/s00221-016-4841-z

**Published:** 2016-11-24

**Authors:** Elena Shumskaya, Marcel A. J. van Gerven, David G. Norris, Pieter E. Vos, Roy P. C. Kessels

**Affiliations:** 1 Donders Institute for Brain, Cognition and Behaviour, Radboud University, Nijmegen, The Netherlands; 2 Erwin L. Hahn Institute for Magnetic Resonance Imaging, Essen, Germany; 3 MIRA Institute for Biomedical Technology and Technical Medicine, University of Twente, Enschede, The Netherlands; 4 Department of Neurology, Slingeland Hospital, Doetinchem, The Netherlands; 5 Department of Neurology, Radboud University Medical Center, Nijmegen, The Netherlands; 6 Department of Medical Psychology, Radboud University Medical Center, Nijmegen, The Netherlands; 7 Radboud University, Donders Centre for Cognition, Montessorilaan 3, 6525 HR Nijmegen, The Netherlands

**Keywords:** Traumatic brain injury, Neuropsychology, Resting-state fMRI, Functional connectivity, Independent component analysis

## Abstract

The aim of this study was to explore modifications of functional connectivity in multiple resting-state networks (RSNs) after moderate to severe traumatic brain injury (TBI) and evaluate the relationship between functional connectivity patterns and cognitive abnormalities. Forty-three moderate/severe TBI patients and 34 healthy controls (HC) underwent resting-state fMRI. Group ICA was applied to identify RSNs. Between-subject analysis was performed using dual regression. Multiple linear regressions were used to investigate the relationship between abnormal connectivity strength and neuropsychological outcome. Forty (93%) TBI patients showed moderate disability, while 2 (5%) and 1 (2%) upper severe disability and low good recovery, respectively. TBI patients performed worse than HC on the domains attention and language. We found increased connectivity in sensorimotor, visual, default mode (DMN), executive, and cerebellar RSNs after TBI. We demonstrated an effect of connectivity in the sensorimotor RSN on attention (*p* < 10^−3^) and a trend towards a significant effect of the DMN connectivity on attention (*p* = 0.058). A group-by-network interaction on attention was found in the sensorimotor network (*p* = 0.002). In TBI, attention was positively related to abnormal connectivity within the sensorimotor RSN, while in HC this relation was negative. Our results show altered patterns of functional connectivity after TBI. Attention impairments in TBI were associated with increased connectivity in the sensorimotor network. Further research is needed to test whether attention in TBI patients is directly affected by changes in functional connectivity in the sensorimotor network or whether the effect is actually driven by changes in the DMN.

**Electronic supplementary material:**

The online version of this article (doi:10.1007/s00221-016-4841-z) contains supplementary material, which is available to authorized users.

## Introduction

Traumatic brain injury (TBI) is a major cause of mortality and morbidity. In Europe, TBI accounts for the greatest number of total years lived with disability resulting from trauma, and it is among the top three causes of injury-related medical costs to society (Maas et al. 2008). Typically, TBI patients suffer from deficits in attention and information processing speed (Merkley et al. 2013; Shumskaya et al. 2012), as well as in executive function and memory (Stuss 2011; Vakil 2005). The most significant challenges for finding effective ways to improve outcomes after TBI are the heterogeneity of the injury and outcomes (Schneider et al. 2014; Dahdah et al. 2014) and identification and classification of patients who would benefit from specific treatments (Saatman et al. 2008).

In addition to focal lesions, diffuse axonal injury (DAI) occurs in TBI, affecting the large white-matter trajectories. As a result, interactions between spatially distinct brain regions become compromised, disrupting cognitive processing (Sharp et al. 2014). This disruption can be studied by two neuroimaging approaches. First, abnormalities in structural connectivity can be investigated using diffusion tensor imaging (DTI), which is sensitive to microstructural white-matter injury (Xiao et al. 2015). Second, alterations in functional connectivity (FC) can be studied by resting-state fMRI. The brain shows spontaneous low-frequency neuronal fluctuations that are synchronized over spatially distributed networks, even in the absence of a specific task. These fluctuations can be measured as the blood oxygen level-dependent (BOLD) response during rest with fMRI (Biswal et al. 1995). The temporal correlation of the time courses between brain regions provides a measure of FC (Hayes et al. 2016). Several resting-state networks (RSNs) have been identified that can be linked to higher-order cognitive processing using rs-fMRI, including the default-mode network (DMN), sensorimotor network, posterior visual processing network, and dorsal attention network (Damoiseaux et al. 2006; Lee et al. 2012).

The most widely studied RSN in TBI is the DMN, a network that is deactivated during controlled cognitive processing. Studies on DMN abnormalities in TBI typically show an increased FC in this network, although some studies also reported a decrease (see Sharp et al. 2014, for an overview). The FC of other networks has typically been found to be decreased, correlating with long-term outcome, including cognitive dysfunction (Xiao et al. 2015). Furthermore, abnormal interactions between the DMN and other RSNs have also been reported in TBI (Sharp et al. 2011). Resting-state fMRI gives new opportunities in TBI diagnostics. That is, it is not only a tool to investigate the connectivity of large-scale RSNs that can be disrupted due to DAI, but also makes it possible to relate this FC to cognitive dysfunction and other outcome measures after TBI (Hayes et al. 2016; Xiao et al. 2015).

In the present study, we hypothesized that (1) TBI influences higher-order cognitive RSNs (including the DMN) and that (2) alterations in functional networks are related to cognitive deficits related to attention and information processing speed.

## Methods

### Participants

TBI patients were selected through the head injury database of the Department of Neurology of Radboud University Medical Centre (Radboudumc), a level I trauma centre. We recruited 47 patients of 18 to 65 years old in the chronic phase of moderate/severe TBI (>1 year post-injury). Moderate TBI was defined as a traumatic injury to the head resulting in a Glasgow Coma Scale (GCS) of 9–12 at the moment of admission, and severe TBI was defined as an injury resulting in a loss of consciousness and GCS ≤ 8 at admission. In addition to the standard MRI contraindications (pacemaker, metal fragments in the body, epilepsy, claustrophobia, pregnancy), the following exclusion criteria were applied: (a) penetrating injury to the skull; (b) a history of severe neurological or somatic disease; (c) psychiatric diagnosis (current and past); (d) neurosurgical operations in past; and (e) severe physical disability or communication deficits that would make a neuropsychological assessment not possible. Three TBI patients were excluded from further analyses due to excessive movement (>3 mm) in the MRI scanner and one patient was excluded because of incomplete MRI scan. At the end, 43 TBI patients were included in the study.

In addition, we recruited 34 healthy control participants (HC) matched in terms of age, sex, and educational level. The same exclusion criteria plus one additional (a history of head injury) were applied to the control participants. Each participant visited the Radboudumc twice. During the first 2-h visit, the neuropsychological test battery (for all participants) and the neurological examination (only for patients) were performed. During the second visit, the participant underwent the MRI scan for approximately 1 h. Both visits were conducted within 1 week.

The study was approved by the Medical Ethics Committee region Arnhem-Nijmegen (CMO registration number 2010/343). All participants gave written informed consent according to the Declaration of Helsinki.

### Functional outcome and neuropsychological assessment

In both groups, mood was assessed using the Beck Depression Inventory (BDI; Beck et al. 1961) to make sure none of the participants had severe depressive symptoms (i.e. BDI > 21, indicative for depression). The Rivermead Post-Concussion Symptoms Questionnaire (RPQ; King et al. 1995) was administered in both groups, consisting of physical, cognitive, and behavioural symptoms that are characteristic for symptomatology in TBI, but which may also be present to some extent in the general population (two subscales, RPQ-3 and RPQ-13). The Glasgow Outcome Scale-Extended (GOS-E) was administered in the TBI group to classify the outcome of each individual patient (Wilson et al. 1998).

The neuropsychological test battery was administered in the patients and HC by two trained neuropsychologists. The assessment consisted of Dutch-language versions of widely used, sensitive and validated neuropsychological tests, covering all major cognitive domains. Verbal and visuospatial episodic memory was assessed with the immediate and delayed recall scores from the Rey Auditory Verbal Learning Test (Van der Elst et al. 2005), the Story recall subtest from the Rivermead Behavioural Memory Test—Third Edition (Wester et al. 2013), and the Location Learning Test-Revised (Bucks et al. 2011). Executive functions were measured using the Brixton Spatial Anticipation Test (rule detection and shifting; Burgess and Shallice 1996), the interference score from the Trail Making Test (mental flexibility) (Bowie and Harvey 2006), the interference score from the Stroop Colour–Word Test (response inhibition) (Van der Elst et al. 2006), and the Letter–Number Sequencing subtest from the Wechsler Adult Intelligence Test-Third Edition (updating/working memory) (Wechsler 1997). Attention was assessed by the Paced Serial Addition Task (2.8 and 2.0 inter-stimulus interval subtests) (Koerts et al. 2012) and the Alertness subtest from the computerized Test of Attentional Performance (Zimmermann and Fimm 2002). The language domain was measured by the letter fluency test (‘D-A-T’; Schmand et al. 2008) and the Boston Naming Task-short version (Van Loon-Vervoorn and Van der Velden 2006). Premorbid verbal intelligence (estimated IQ) was assessed using the National Adult Reading Test (Schmand et al. 1992).

Raw scores were converted into standardized *Z*-scores in order to directly compare the individual tests and to group them into cognitive domains. These *Z*-scores were calculated on the mean and pooled standard deviations of the whole group taken together. *Z*-scores were reversed for reaction times and interference scores, resulting in higher scores representing a better performance for all measures subsequently. Composite domain scores were calculated for the domains described above by computing the mean of the *Z*-scores of the individual tests comprising that domain. The composite domain scores were used as outcome measures (Brands et al. 2007). We used ANOVAs to explore the differences between groups.

### MRI data acquisition

Patients and controls underwent the same imaging protocol on a 3 Tesla Siemens Trio scanner (Erlangen, Germany) with a 32-channel head coil. Resting-state data were acquired by using a multiecho EPI with the following parameters: TR = 2000 ms, TEs = 6.9/16.2/25.0/35.0/44.0 ms, voxel size = 3.5 × 3.5 × 3.0 mm, 800 volumes (Poser et al. 2006). Participants were instructed to relax with open eyes in complete darkness.

To obtain a high-resolution structural T1-weighted image, a volumetric magnetization prepared rapid gradient echo (MPRAGE) sequence with the following parameters was used: TE/TR/TI = 2.94/2300/1100 ms, flip angle = 8°, voxel size = 1.0 × 1.0 × 1.0 mm.

### MRI preprocessing

Image preprocessing was performed using FSL software (http://fmrib.ox.ac.uk/fsl). Preprocessing included deleting the first five volumes to allow the magnetization to reach dynamic equilibrium, and retaining the subsequent 795 volumes, motion correction with MCFLIRT, removal of non-brain tissue, grand mean scaling to normalize the global 4D data, and spatial smoothing using a Gaussian kernel of 6 mm full width at half-maximum. Subsequently, nuisance regression was performed using the approach, commonly approved by scientific community for careful reducing motion-induced artefacts (Satterthwaite et al. 2013). This regression removed signals from the white matter (WM) and cerebrospinal fluid (CSF) as well as 24 motion parameters. WM and CSF signals were derived from the masks created using FSL FAST. Six motion parameters (three translations and three rotations) were derived from the motion correction procedure, as well as the temporal derivatives of these six parameters plus six frame-to-frame parameters (i.e. the motion in one frame relative to the previous frame) and the temporal derivatives of those, resulting in 24 parameters. Lastly, a high-pass temporal filter was used with a cut-off of 100 s. The preprocessed functional images were linearly registered with FLIRT to the subject-specific high-resolution T1 images using boundary-based registration transformation (Greve and Fischl 2009). Subsequently, these images were registered to MNI standard space using 12-parameter affine transformation and nonlinear registration with FNIRT (10-mm warp, 4-mm resampling resolution; Jenkinson et al. 2002).

### Resting-state connectivity analysis

We conducted group independent component analysis (ICA) using MELODIC (Beckmann et al. 2005). Ten independent components (ICs) were selected as corresponding to major RSNs. These included three visual, two sensorimotor, auditory, posterior part of the default-mode network (DMN), executive control, right and left frontoparietal networks. Furthermore, we selected five additional ICs that resembled RSNs, but did not show high correlation with the previously reported networks (Smith et al. 2009). These were the anterior part of the DMN, two cerebellar, frontal pole, and thalamus networks. To investigate FC patterns of each participant for each IC, we employed a dual regression approach (Filippini et al. 2009). Further details are provided in the supplemental document.

This analysis generated spatial maps for each RSN indicating between-group differences. To control for false positives introduced by investigating all 15 RSNs, effects were significant if they reached the two-tailed *p* values <0.002 (family-wise error (FWE) corrected). However, as this correction is very conservative and because of the nature of the analyses, we also report effects with a *p* value <0.05 (FWE corrected) and with a minimum of five voxels. MNI coordinates of peak voxels were linked to anatomical locations using the Harvard–Oxford cortical and subcortical atlases and the cerebellum atlas in MNI152 space that are implemented in FSL.

### Relationship of functional connectivity measures to neuropsychological scores

To investigate the relationship between connectivity strength and the cognitive domain scores, we performed multiple linear regression analysis. Connectivity values (mean *Z*-score from the second stage of the dual regression) were extracted from the clusters that showed a significant effect in the group analysis. The cognitive domain scores served as dependent variables, whereas the group variable (patient/control), connectivity scores from five RSNs, and five interactions ‘group-by-RSN connectivity’ were taken as independent variables with correction for nuisance covariates (age, sex, and education).

## Results

We compared FC in 43 TBI patients with 34 HC. These groups did not differ in age, sex distribution, educational level, and handedness (Table 1). The groups were also well matched with respect to IQ. According to GOS-E, most of the TBI patients (93%) showed moderate disability. No differences were found with respect to depressive symptoms between TBI patients and HC.

**Table 1:** Demographic and outcome information of moderate/severe TBI patients and healthy controls

	TBI (*n* = 43)	Controls (*n* = 34)	Statistic	*p* value
*Demographic*				
Age (years)	42.3 (14.9; 19–65)	44.9 (12.9; 18–63)	*t*(75) = 0.81	0.42
Sex (males)	25 (58%)	20 (59%)	*χ* ^2^(1) = 0.004	0.95
Educational level			*U* = 711.5, *Z* = −0.22	0.83
Primary school, no further education	1 (2%)	0 (0%)		
More than primary school, no diploma	0 (0%)	0 (0%)		
Lower secondary education	5 (12%)	3 (9%)		
Average secondary education	22 (51%)	19 (56%)		
Higher secondary education	11 (26%)	10 (29%)		
Academic degree	4 (9%)	2 (6%)		
NART IQ	91.9 (13.3; 73–128)	94.5 (8.679–112)	*t*(74) = 0.97	0.33
Handedness (right)	40 (93%)	26 (77%)		0.06
*Outcome*				
Time since accident (months)	80.1 (43.7; 21–160)			
GCS	5.8 (3.4; 3–12)			
GOS-E				
4 (Upper severe disability)	2 (5%)			
5 (Lower moderate disability)	27 (63%)			
6 (Upper moderate disability)	13 (30%)			
7 (Lower good recovery)	1 (2%)			
RPQ-3	1.4 (2.3; 0–9)	0.4 (1.1; 0–4)	*t*(63.0) = 2.6	0.024
RPQ-13	11.6 (11.3; 0–40)	4.3 (5.6; 0–19)	*t*(64.9) = 3.7	<0.001
BDI	5.2 (5.2; 0–21)	4.4 (5.2; 0–17)	*t*(65.2) = 0.7	0.51

Mean (standard deviation; range) or *n* (%) are presented

*BDI* Beck Depression Inventory, *GCS* Glasgow Coma Scale, *GOS-E* Glasgow Outcome Scale-Extended, *NART* National Adult Reading Test, *RPQ* Rivermead Post-Concussion Symptoms Questionnaire

A trend towards more head motion during the scan was found in the TBI group, but this did not reach the significance threshold (root mean square of relative motion was 0.042 (SD = 0.020) mm in TBI and 0.033 (SD = 0.015) mm in HC; *p* = 0.051). The root mean square of relative motion (frame-to-frame parameter) was carefully regressed out during the motion correction procedure (see section on “MRI preprocessing”).

### Neuropsychological performance

**Fig. 1:**
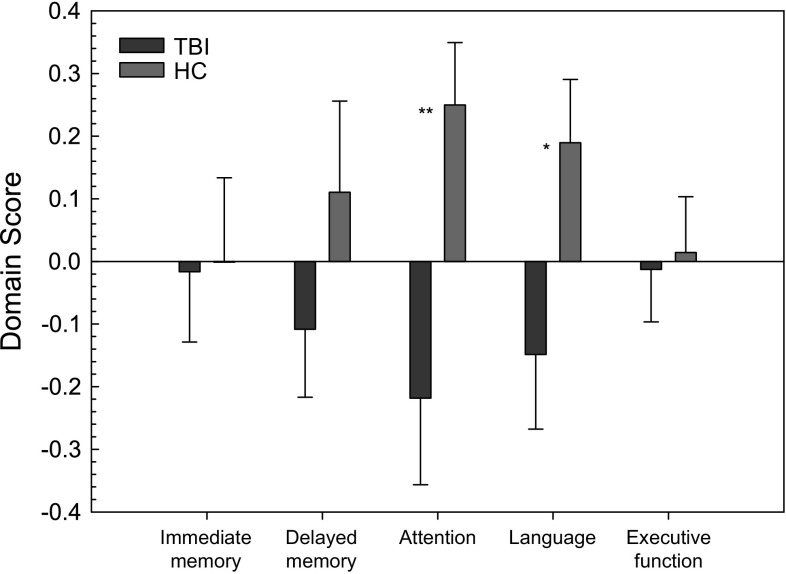
Group comparison of neuropsychological outcome in traumatic brain injury (TBI) patients and healthy controls (HC); mean (SE) for each cognitive domain are presented (*p = 0.039; **p = 0.011)

A strong group effect showing a worse cognitive performance in the TBI group was found for the attention and language cognitive domain scores [*F*(1,75) = 6.848, *p* = 0.011 and *F*(1,75) = 4.406, *p* = 0.039, respectively; see also Fig. 1]. No main effect of group was found for the other cognitive domains (all *p* values <1.518). Because the composite scores of domains attention and language were assessed by means of two subtests in each domain, we explored further the differences between the groups in each of these subtests. The TBI group showed significantly slower reaction times on the TAP Alertness subtest [mean *Z*-score (standard error) = −0.29 (0.18) in TBI and 0.36 (0.11) in HC; *p* = 0.004] and a worse performance on the Boston Naming Task [mean *Z* TBI: −0.21 (0.18); HC: 0.26 (0.70); *p* = 0.04]. No significant group differences were found between the TBI group and HC on the PASAT or verbal fluency test.

### Altered resting-state FC in TBI

Stronger connectivity in the TBI group was found in five RSNs: the sensorimotor, visual, the posterior part of the DMN, executive control, and cerebellum. Effects in the cerebellum and visual RSNs survived thresholding at *p* < 0.002 (FWE), while others survived at *p* < 0.05 (FWE, size ≥5 voxels).

**Fig. 2:**
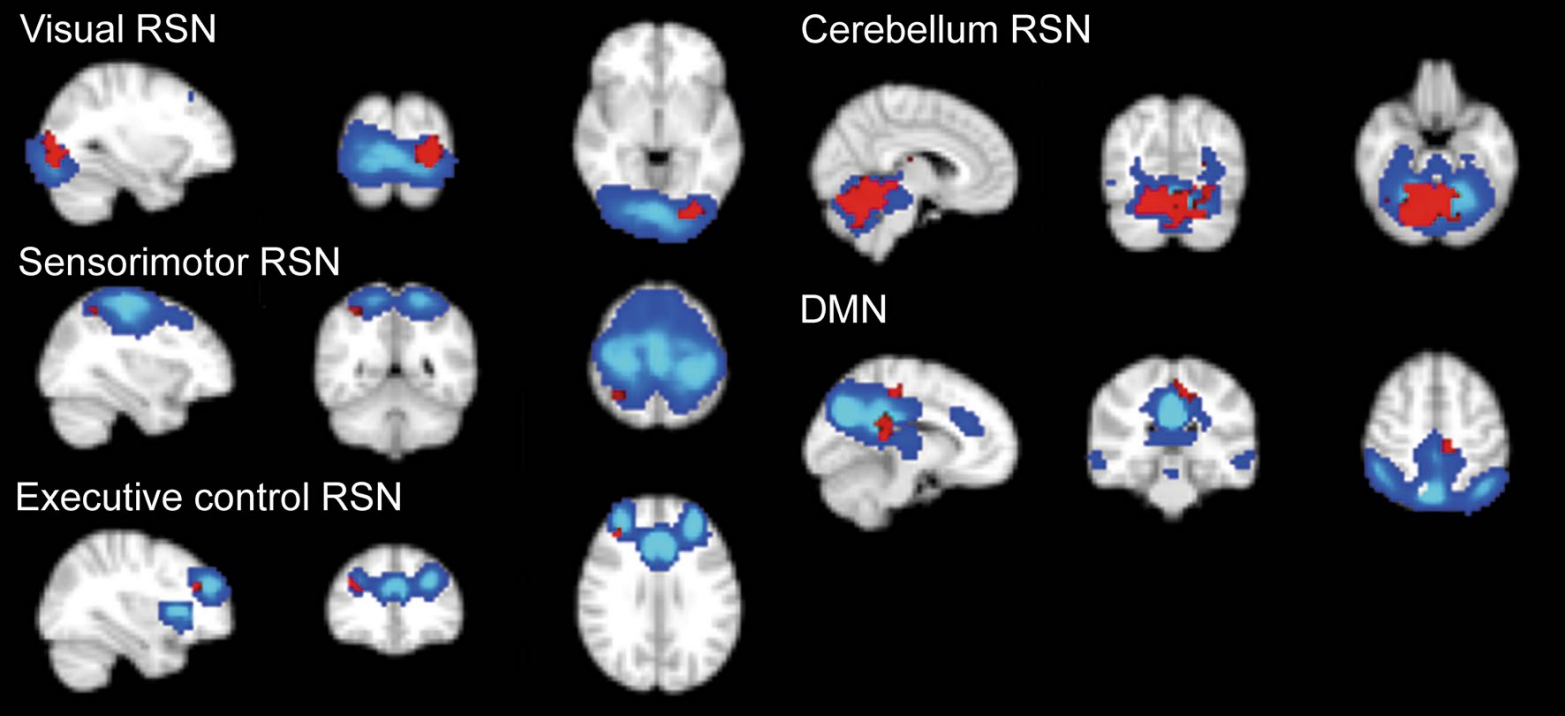
Between-group differences in resting-state networks (RSN). *Cold colours* represent the spatial map of the corresponding network (the *Z*-score colour scale is from 5 to 30), *red colour* represents the clusters showing the differences between TBI patients and healthy controls. The *left side of the brain* corresponds to the *right side in the image*. *DMN* default-mode network (colour figure online)

The clusters of stronger connectivity were located in the occipital fusiform gyrus L (visual RSN), vermis VI (cerebellum), superior parietal lobule R (sensorimotor RSN), precentral and posterior cingulate gyri L (DMN), and middle frontal gyrus R (executive control RSN) (Fig. 2).

We did not find stronger connectivity for HC versus TBI in any of the 15 RSNs.

### Relationship of FC measures to neuropsychological scores

**Fig. 3:**
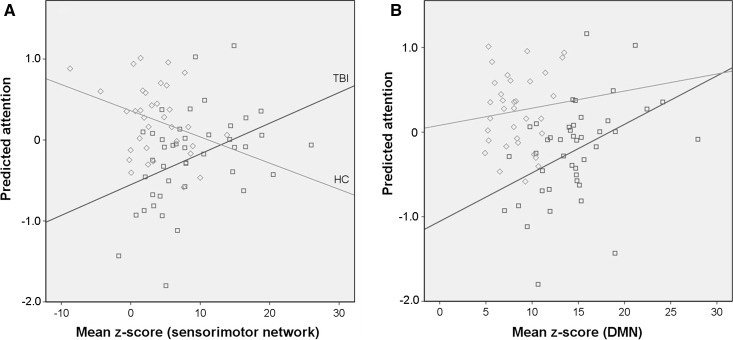
Relation between functional connectivity strength and attention score in sensorimotor network (**a**) and the default-mode network (DMN) (**b**). Mean *Z*-score is derived by averaging within the clusters showing the differences between the traumatic brain injury patients (TBI) and healthy controls (HC) in the corresponding RSN. Unstandardized predicted value for attention is the output of the linear regression where the group variable, mean *Z*-scores from five RSNs, and five interactions between group variable and *Z*-scores are taken as independent variables with correction for nuisance covariates (age, sex, and education). TBI group is in blue, HC group is in green. **a** *R* ^2^ linear = 0.172 (TBI), *R* ^2^ linear = 0.095 (HC) and **b** *R* ^2^ linear = 0.185 (TBI), *R* ^2^ linear = 0.011 (HC)

To investigate the relation between the attention deficits and the abnormal FC, we performed multiple linear regression analyses. As expected from the analysis of the neuropsychological outcome, which revealed a lower performance in attention in TBI, the effect of the group variable in multiple linear regression was significant (Table 2). We found a significant effect of the connectivity in sensorimotor RSN on attention (*p* < 10^−3^) and a trend to significant effect of the connectivity in DMN (*p* = 0.058). A ‘group-by-RSN’ interaction on attention was found in the sensorimotor network (*p* = 0.002). In TBI patients’, attention was positively related to the resting-state connectivity in sensorimotor RSN, while in HC the relation was negative (Fig. 3a). For the DMN, no significant ‘group-by-RSN’ interaction in attention was found (*p* = 0.198, Fig. 3b).

**Table 2:** Relationship between attention and functional connectivity in clusters showing differences between the traumatic brain injury patients and the healthy controls expressed as the output of linear regression where group, mean *Z*-scores from clusters in five RSNs, and five interactions between group variable and *Z*-scores are taken as independent variables with correction for nuisance covariates (age, sex, and education)

	Unstandardized beta	*p* value
*Independent variable*		
Group	0.62	0.034*
Sensorimotor RSN	0.20	<10^−3^**
DMN	0.18	0.055
Executive control RSN	−0.04	0.434
Visual RSN	0.03	0.489
Cerebellum RSN	−0.08	0.124
Group × sensorimotor RSN	−0.12	0.002**
Group × DMN	−0.09	0.198
Group × executive control RSN	0.04	0.228
Group × visual RSN	−0.03	0.374
Group × cerebellum RSN	0.03	0.482

Adjusted *R* ^2^ = 0.371

* *p* < 0.05; ** *p* < 0.005

The relationship between the sensorimotor network and DMN was also explored and found to be different within the TBI and HC groups. In the TBI patients, the connectivity in the sensorimotor network was positively correlated with the DMN connectivity (Pearson correlation coefficient = 0.441, *p* = 0.003). In HC, this relation showed a trend towards the opposite direction (i.e. a higher DMN connectivity was related to a lower connectivity in the sensorimotor network), although this effect was not significant.

## Discussion

We report stronger FC during resting state in chronic moderate/severe TBI patients in a number of RSNs: sensorimotor, DMN, visual, executive control, and cerebellar RSNs. This suggests that TBI affects higher-order cognitive networks (DMN, executive control, cerebellum) as well as sensory networks (sensorimotor, visual). A stronger function coupling within these networks may underlie behavioural impairments and symptoms observed in TBI and/or be indicative of compensation mechanisms, leading to recovery of impaired cognitive functions.

We provided evidence for a significant effect of altered functional connectivity in the sensorimotor RSN on attention impairments after TBI. In particular, a test for information processing speed (TAP Alertness) turned out to be the most sensitive test, showing significantly slower reaction times in TBI patients, which is one of the core cognitive deficits after TBI (Willmott et al. 2009). In this subtest, the participant must respond to a stimulus and increase his/her attentional level in expectance of a stimulus of high priority. Importantly, the relation between FC in the sensorimotor RSN and attention was opposite in TBI and HC. We found a positive correlation between FC and attention in the TBI group, showing that in patients with attention deficits as FC in sensorimotor network increases, the attentional performance also increases.

At the same time, we observed a negative correlation between connectivity in the sensorimotor network and attention in HC. The possible explanation of this relation might be as follows. While sensorimotor RSN is classified as a lower-level sensory network, it might be considered a task-positive network that is activated during the task and suppressed at rest when the task-negative DMN is prominent. In the HC group during resting-state fMRI, the sensorimotor network has been suppressed to keep the balance with the DMN activation, and this fact might help to understand a negative relation between resting-state FC in sensorimotor network and the attention score in HC (cf. Sharp et al. 2014).

We also found a differential coupling between the sensorimotor RSN (as a lower-level task-positive network) and the DMN in TBI and HC. The relationship between these two networks was not significant in HC, but showed a negative trend. In TBI patients, both networks were positively correlated, indicating that the relation between task-positive sensorimotor network and task-negative DMN is impaired.

Furthermore, we were interested in evaluating FC within the DMN in TBI because recent studies (Bonnelle et al. 2011; Sharp et al. 2011) reported a relationship between changes in the DMN FC and attention deficits after TBI. Similarly to these previous reports, we observed an increase in FC within the DMN in TBI which has been related to impairments in attention. We investigated further the abnormal patterns of the DMN FC in relation to attentional performance. Here, we observed a trend (*p* = 0.058) towards a positive relation between the resting-state FC in DMN and attention in both groups. However, the TBI group showed a stronger effect, meaning that abnormally high connectivity of precentral and posterior cingulate gyri within the DMN correlated with more efficient response speed. This finding is in line with a previously stated hypothesis (Bonnelle et al. 2011), suggesting that such modification within the DMN is a novel mechanism for attention recovery after TBI.

Altogether, our findings suggest that the coupling between the DMN and the task-positive sensorimotor network is impaired in TBI. Increased FC in five RSNs might be a sign of a higher cognitive load in TBI patients. It seems the mechanisms of relationships between the sensorimotor FC and attention might involve the coupling with the DMN and probably are of different nature in TBI and HC groups. However, in order to test this hypothesis further research is needed, which would clarify whether the attention in TBI patients is directly affected by abnormal FC in the sensorimotor network or it is rather an epiphenomenon and the effect is actually driven by DMN changes. Another intriguing question would be how this effect behaves in the milder form of TBI.

Some methodological issues should be mentioned here. Our interpretations are based on cross-sectional data; therefore, no causal inferences can be made. Future longitudinal studies are needed to better understand the temporal evolution of FC related to TBI (Xiao et al. 2015). Furthermore, according to the neuropsychological assessment, the cognitive performance of TBI patients included in our study was comparable to HC in most cognitive domains. Although all patients were classified (using GCS) as moderate to severe TBI and 98% showed moderate to upper severe disability based on the GOS-E, the design of our study may have resulted in a bias towards a good cognitive outcome, as we only recruited patients in the chronic stage. As a result, we should be cautious in generalizing our findings of a relationship between attention deficits and increased FC in sensorimotor RSN and DMN to TBI patients with more severe symptoms, for instance, in the subacute stage.

A potential confound that should be considered in the present study is the trend towards increased motion during MRI acquisition in patients compared to healthy controls. One might argue that increased connectivity in the sensorimotor network in the patient group could be attributed to subject movement. However, it was found that connectivity in the sensorimotor network in patients was positively correlated with attention. This is contrary to the hypothesis that more attentionally impaired patients are expected to move more. Nevertheless, these and other potential confounds should be taken into account given their known impact on functional connectivity estimates (van Dijk et al. 2012).

Finally, until recently, most studies measured FC at rest by detecting the temporal correlations of spontaneous fluctuations in BOLD. It is implicitly assumed that FC was stationary during acquisition. These approaches do not consider temporal variations in FC, providing an average over the acquisition period. Recently, it has been increasingly recognized that FC is dynamic in nature. For example, clustering analysis of human brain networks displayed dynamic but quasi-stable connectivity patterns that diverged from the averaged connectivity pattern (Allen et al. 2014). This finding presents a new challenge but also provides new perspectives for understanding human brain networks and how FC is modified by different diseases, including TBI.

In conclusion, our study in chronic moderate to severe TBI patients demonstrates an increase in FC within both sensory RSNs (sensorimotor and visual) and higher-order cognitive networks (DMN, executive, cerebellum). We provide strong evidence on the relation between abnormal FC in the sensorimotor RSN and attentional performance. Moreover, this relation is opposite in TBI patients with attention deficits and HC. We found a trend towards a positive correlation between abnormal FC within the DMN and attention. Further research is needed for better understanding of the temporal evolution of FC abnormalities and their effects on cognitive outcome.

## Electronic supplementary material

Below is the link to the electronic supplementary material.

Supplementary material 1 (DOCX 12 kb)

## Abbreviations

DMN: Default-mode network
fMRI: Functional magnetic resonance imaging
ICA: Independent component analysis
RSN: Resting-state network
TBI: Traumatic brain injury
