# Supplementary material for: Abnormal connectivity in the sensorimotor network predicts attention deficits in traumatic brain injury
Source: Exp Brain Res. 2016 Nov 24;235(3):799–807. doi: 10.1007/s00221-016-4841-z (PMC5315712; doi:10.1007/s00221-016-4841-z)
Supplement: Supplementary file 1 — Supplementary material 1 (DOCX 12 kb) [file 221_2016_4841_MOESM1_ESM.docx]

**Supplemental document**

**Independent Component Analysis**

We conducted group Independent Component Analysis (ICA) using MELODIC in FSL (Beckmann et al., 2005). Functional images of all participants were concatenated in the temporal domain to create a single 4D dataset. This concatenated dataset was then decomposed into 30 spatially independent components (ICs). These 30 components were compared with maps of previously identified RSNs, the ten maps from Smith et al. (2009) by means of spatial correlation using the FSL utility function ‘fslcc’. ICs that showed a spatial correlation above 0.4 with a previously defined RSN were selected and further visually inspected to check that both the spatial and temporal pattern represented a functional network. Based on these criteria we selected ten ICs as corresponding to these major RSNs. These included three visual, two sensorimotor, auditory, default mode, executive control, right- and left frontoparietal networks. Furthermore, we selected five additional ICs that did resemble RSNs, but did not show high correlation with the Smith RSNs. These were the default mode, two cerebellum, frontal pole, and thalamus networks.

**Resting-state connectivity analysis using dual regression**

To investigate functional connectivity patterns of each participant for each IC we employed a dual regression approach (Filippini et al., 2009). Using the 30 maps from the group ICA as ‘templates’ we conducted two separate regressions. In the first regression the 30 spatial maps were used as regressors against the preprocessed individual subjects’ fMRI data. This produced subject-specific time courses corresponding to each IC. In the second regression these time courses were variance-normalized and used as regressors against the individual subjects’ fMRI data to produce participant-level unique spatial maps for each of the 30 ICs. This way, the subject-specific spatial maps reflect the relationship between an individual voxel’s timecourse and the IC time course, thus representing the participation of each voxel in the network (Janes et al., 2012). Both the first and second regressions were multiple regressions, i.e. all spatial maps or time courses were entered as simultaneous regressors in a single regression model.

To identify group differences within the 15 components of interest described above, the 15 corresponding participant-level spatial maps were concatenated across subjects into 4D files and tested voxel-wise for significant differences between TBI patients and the healthy controls via a general linear model. For this we employed non-parametric permutation testing (applying 5000 permutations) with threshold-free cluster enhancement (TFCE, (Smith and Nichols, 2009)) using the Randomize tool of FSL. Gender, age, education, handedness, and mean root mean square (rms) relative motion were added to the model as covariates of no interest. The rms relative (frame-to-frame) motion was computed with MCFLIRT at the motion correction stage during preprocessing (Jenkinson et al., 2002) and was averaged over all volumes to obtain a single measure of head motion per participant. This motion parameter was chosen as it was previously reported that using average frame-wise displacement as a covariate on the group level reduces motion-related artifacts (Fair et al., 2013).

This analysis generated spatial maps for each RSN indicating between-group differences. To control for false positives introduced by investigating all 15 RSNs, effects were significant if they reached the two-tailed p-values < 0.002 (family-wise error (FWE) corrected). However, as this Bonferroni correction is very conservative and the nature of the analyses is exploratory, we also report effects with a p-value < 0.05 (FWE corrected) and with a minimum of 5 voxels. Montreal Neurological Institute (MNI) coordinates of peak voxels were linked to anatomical locations using the Harvard-Oxford cortical and subcortical atlases and the cerebellum atlas in MNI152 space that are implemented in FSL.
